# Antibodies directed against extracellular loops of FadL orthologs disrupt outer membrane integrity and neutralize infectivity of *Treponema pallidum*, the syphilis spirochete

**DOI:** 10.3389/fimmu.2025.1724458

**Published:** 2026-01-02

**Authors:** Kristina N. Delgado, Crystal F. Vicente, Carson J. La Vake, Everton Bettin, Melissa J. Caimano, Justin D. Radolf, Kelly L. Hawley

**Affiliations:** 1 Department of Pediatrics, UConn Health, Farmington, CT, United States; 2 Department of Medicine, UConn Health, Farmington, CT, United States; 3 Department of Molecular Biology and Biophysics, UConn Health, Farmington, CT, United States; 4 Department of Research, Connecticut Children’s Research Institute, Hartford, CT, United States; 5 Department of Immunology, UConn Health, Farmington, CT, United States; 6 Department of Genetics and Genome Sciences, UConn Health, Farmington, CT, United States; 7 Division of Infectious Diseases and Immunology, Connecticut Children’s, Hartford, CT, United States

**Keywords:** syphilis, *Treponema pallidum*, FadL, extracellular loop, outer membrane damage, growth inhibition

## Abstract

A vaccine is urgently needed to curtail the global epidemic of syphilis, a sexually transmitted infection caused by the spirochetal pathogen *Treponema pallidum* (*TPA*). Protective antibodies must target extracellular loops (ECLs) of *TPA* outer membrane proteins (OMPs). Immune rabbit serum (IRS) and antibodies elicited by immunization with a *Pyrococcus furiosus* thioredoxin (*Pf*Trx) scaffold displaying ECLs from BamA (TP0326) and three FadLs (TP0856, TP0858 and TP0865) inhibited growth and metabolic activity of spirochetes during *in vitro* cultivation. Flow cytometric analysis of GFP-expressing *TPA* using the membrane impermeable dye propidium iodide revealed that IRS and growth-inhibiting ECL-specific antibodies disrupted the spirochete’s fragile outer membrane. Spirochetes incubated with IRS or growth-inhibiting ECL-specific antibodies produced no or only transient lesions with markedly reduced bacterial burdens following intradermal inoculation into rabbits, confirming neutralization of infectivity. This study advances efforts to define immune correlates of protection to guide rational development of a multivalent, ECL-based syphilis vaccine.

## Introduction

Syphilis, caused by the sexually transmitted, obligate human pathogen *Treponema pallidum* subspecies *pallidum* (*TPA*), remains a major global health challenge (1, 2). With over seven million new cases reported annually and rising incidences worldwide (1–4), the need for a vaccine with global efficacy is more urgent than ever (5, 6). Antibodies play a central role in vaccine-induced protective immunity against extracellular pathogens by specifically targeting surface-exposed epitopes (7–9). In the case of *TPA*, also an extracellular pathogen (10–12), investigators in the pre-molecular era generated compelling *in vitro* (13–15) and *in vivo* (13, 16) evidence that antibodies play a pivotal role in spirochete clearance and protective immunity. With the onset of the molecular era, identification of potentially protective surface-exposed antigens was hampered by the spirochete’s fragile outer membrane (OM) and the structure’s atypical molecular architecture, most notably its low density of integral OM proteins (OMPs) (17–19). However, it is now well established that *TPA*’s repertoire of rare OMPs, termed the *TPA* OMPeome, constitutes the principal reservoir of candidate vaccinogens (17, 20–23). Similar to other diderm bacteria (24, 25), OMPs in *TPA* adopt β-barrel structures with extracellular loops (ECLs) as the antibody accessible regions (21, 26, 27). To identify ECLs that elicit functional, potentially protective antibodies, our group developed a novel platform using a thermostable *Pyrococcus furiosus* thioredoxin (*Pf*Trx) scaffold (28) to display individual ECLs in a conformationally constrained form (**Supplementary Figure S1A**) (29–31). This strategy not only facilitated the identification of promising ECL vaccine candidates but also provided new insights into the mechanisms whereby surface-directed antibodies promote spirochete clearance during infection and mediate protection following immunization.

A critical step in vaccine development is establishing an *in vitro* correlate of immunity, a measurable immune response that serves as a marker for protection (32–36). To date, no such correlate has been established for syphilis. Historically, antibodies that promote macrophage-dependent phagocytosis *ex vivo* have been considered the primary mechanism for *TPA* clearance *in vivo* (14, 15, 37–39). However, recent advances in long-term *TPA* cultivation (40) combined with our use of scaffolded ECLs, have brought to light a previously unrecognized mechanism for antibody-mediated spirochete clearance. We recently reported that antibodies targeting ECL4 of BamA (TP0326) and ECLs of the FadL orthologs TP0856, TP0858, and TP0865 directly impair spirochete viability *in vitro* (29). Notably, these growth inhibitory effects occurred independently of macrophages or complement, suggesting that ECL-specific antibodies compromise spirochete fitness by disrupting the functions of essential OMPs. In a follow up study, we demonstrated by flow cytometry, using a virulent genetically engineered green fluorescent protein (GFP)-expressing *TPA* strain and the membrane-impermeant fluorescent DNA dye propidium iodide (PI), that antibodies in immune rabbit serum (IRS) and antibodies directed against ECL4 of BamA (TP0326) disrupt OM integrity (11). Building on these findings, we show herein that IRS and growth inhibitory FadL ECL antibodies elicit irreparable OM damage and neutralize spirochete infectivity. In addition to expanding the field’s toolkit to improve understanding of *TPA*’s enigmatic interactions with surface-directed antibodies generated during infection (20, 41, 42), this study advances efforts to define immune correlates of protection to guide rational development of a multivalent, ECL-based syphilis vaccine.

## Methods

### Ethics statement

Animal experimentation was conducted following the *Guide for the Care and Use of Laboratory Animals* (8th Edition) in accordance with protocols reviewed and approved by the UConn Health Institutional Animal Care and Use Committee (AP-200351-0124, AP-200362-0124, AP-201085-1226, and AP-201086-1226) under the auspices of Public Health Service assurance number A3471-01 (D16-00295).

### Immunologic reagents

Immune rabbit serum (IRS) was obtained following intratesticular inoculation of a New Zealand white rabbit with freshly *harvested* rabbit passaged *TPA* (Nichols strain) as previously described (29, 30). Rabbit antisera directed against recombinant TP0751 and *Pf*Trx scaffolds displaying BamA ECL4, TP0856 ECL2 and ECL4, TP0858 ECL2 and ECL4, and TP0865 ECL3 (see schematic in **Supplementary Figure S1A**) were described previously (23, 27, 29, 31). As part of our prior studies, a codon-optimized *Pyrococcus furiosus* thioredoxin (*Pf*Trx) gene containing the *TPA* BamA ECL4 inserted between residues 26 and 27 and a C-terminal Avi-Tag (GLNDIFEAQKIEWHE) was synthesized (Genewiz) and cloned into NdeI–XhoI–digested pET28a by In-Fusion cloning. *Pf*Trx^Empty^ was generated by BamHI digestion of *Pf*Trx^BamA/ECL4^ to remove the ECL4 insert followed by self-ligation. The *Pf*Trx scaffolds displaying FadL ECLs were produced either by inverse PCR with ECL-encoding primers or by inserting PCR-amplified ECLs from synthetic genes into BamHI-digested pET28a*^Pf^*^Trx^ by In-Fusion cloning. Plasmids were then transformed into *E. coli* BL21-Gold (DE3) (Agilent, Santa Clara, CA) for overexpression. All constructs were purified over Ni-NTA resin (Qiagen, Germantown, MD) followed by size exclusion chromatography. Adult male NZW rabbits were sedated with acepromazine (1–2 mg/kg) and then anesthetized with 3–5% isoflurane prior to each immunization. Animals were primed with a total of 200 µg of *Pf*Trx-scaffolded ECL in 500 µL of PBS–TiterMax (1:1, vol/vol), administered as four subcutaneous injections (100 µL each) and two intramuscular injections (50 µL each). Rabbits were boosted at 3, 6, and 9 weeks with the same volume and amount of protein in PBS–TiterMax (1:1, vol/vol). At 12 weeks, animals were given an overdose of a ketamine cocktail (1.5–2.0 mL containing ketamine [25–50 mg/kg], xylazine [2.5–5 mg/kg], and acepromazine [0.75–1 mg/kg]) for sedation. Once adequately sedated, animals were exsanguinated and then pentobarbital (200 mg/kg) was injected intravenously, and a thoracotomy was performed to confirm death.

### *In vitro* cultivation of GFP^+^*TPA*

GFP^+^*TPA* (Nichols) (11) were co-cultured with cottontail rabbit epithelial cells (Sf1Ep) in *TPA* culture medium 2 (TpCM‐2) under microaerophilic conditions as previously described (40, 43). Briefly, Sf1Ep were seeded at 5 x 10^4^ cells per well in a six-well culture plate and incubated overnight at 37 °C. The following day, wells were washed with TpCM‐2 followed by the addition of fresh TpCM‐2 medium, and 5 x 10^6^*TPA* were added per well. After 7 days, and weekly thereafter, treponemes were harvested by trypsinization, enumerated by darkfield microscopy (DFM), and passaged onto freshly seeded Sf1Ep cells. The same procedure was used for *in vitro* cultivation in 24-well plates, with the exception that wells were seeded with 2 x 10^4^ Sf1Ep cells and 2.5 x 10^6^*TPA* were added per well.

### Growth inhibition of GFP^+^*TPA* by IRS and ECL antibodies

As previously described (11, 29) Sf1Ep cells were seeded at 2 x 10^4^ cells/well in a 24-well culture plate and incubated overnight at 37 °C. The following day, 2.5 x 10^6^ freshly harvested *in vitro* cultivated GFP^+^*TPA* were added to each well along with 10% heat-inactivated normal rabbit sera (NRS), IRS (IRS Nic-1), or *Pf*Trx ECL-specific rabbit antisera (*Pf*Trx^TP0856/ECL2^, *Pf*Trx^TP0856/ECL4^, *Pf*Trx^TP0858/ECL2^, *Pf*Trx^TP0858/ECL4^, *Pf*Trx^TP0865/ECL3^). Control antisera included α-*Pf*Trx^BamA/ECL4^ and α-TP0751 (23, 27, 31). Spirochetes were harvested following incubation for seven days under microaerobic conditions for enumeration by DFM (11, 29, 40, 43). Statistical analyses were conducted using Prism v. 9.5.1 (GraphPad Software, San Diego, CA, USA). A two-way ANOVA was used to compare *TPA* growth *in vitro*, with Bonferroni’s correction for multiple comparisons. *p*-values < 0.05 compared to the NRS control were considered significant.

### Fluorescence microscopy of GFP^+^*TPA* co-cultured with NRS, IRS or BamA ECL4 antibodies

GFP^+^*TPA* spirochetes were co-cultured in a 24-well tissue culture plate with Sf1Ep cells and 10% heat-inactivated NRS, IRS, or α-*Pf*Trx^BamA/ECL4^ for 7 days at 37°C under microaerophilic conditions as previously described (11, 29). Treponemes were treated as described below for flow cytometry except that the final pellet was resuspended in 20 µl of PBS. 10 µl of the suspension was transferred to a glass microscope slide and immediately covered with a glass coverslip. Images were acquired using an epifluorescence Olympus BX-41 microscope equipped with 40x Plan-Apochromat objective and processed with VisiView v. 5.0.0.7 (Visitron Systems GmbH, Puchheim, Germany) and ImageJ 1.54g (Wayne Rasband and contributors, National Institutes of Health, USA; http://imagej.org; Java1.8.0_345,64-bit).

### Flow cytometric assessment of OM disruption by PI staining

Samples were processed for flow cytometry and analyzed in triplicate as described previously (11). Briefly, treponemes were pelleted at 8,000 x *g* for 10 minutes, washed twice with phosphate-buffered saline (PBS), and fixed for 10 min at 4 °C in PBS containing 2% paraformaldehyde and 0.001% PI. After fixation, treponemes were pelleted at 8,000 x *g* for 10 minutes and washed twice with PBS; the resulting pellets were resuspended in 200 µl of PBS and transferred to a 96-well plate. 25 µl of each sample was analyzed on a FACSymphony A5 SE flow cytometer (BD Biosciences, Franklin Lakes, NJ, USA). Data were analyzed using FlowJo v10.7.1 (BD Biosciences). Gating strategies used to identify GFP^+^/PI^+^ spirochetes and exclude non-spirochetal events (*i.e*., double negatives) (11), as detailed in the Supplementary Information for each figure, with a representative example shown in **Supplementary Figure S2**. The percentage of PI^+^ organisms within the GFP^+^ population and their mean fluorescence intensity (MFI) were quantified in FlowJo. Integrated MFI (iMFI) for PI staining was calculated by multiplying the frequency of PI^+^ spirochetes by their corresponding MFI values (44). Statistical analyses were performed using Prism v. 9.5.1 (GraphPad Software). Two-way ANOVA with Bonferroni’s correction was used for comparing spirochete counts across groups, and one-way ANOVA with Bonferroni’s correction was used for iMFI comparisons. Differences were considered statistically significant at *p*-values < 0.05.

### Assessment of metabolic activity of GFP^+^*TPA* using alamarBlue

GFP^+^*TPA* were co-cultured with Sf1Ep cells in a 24-well tissue culture plate in the presence of 10% heat-inactivated NRS, IRS, or ECL-specific polyclonal antisera, and incubated under microaerophilic conditions. After seven days, GFP^+^*TPA* was harvested and enumerated by DFM as described above. For assessment of metabolic activity, treponemes from each condition were normalized to 1 × 10^4^ GFP^+^*TPA* in 100 μl of TpCM-2 medium and transferred to a black-walled, clear-bottom 96-well imaging plate (Sigma-Aldrich, Burlington, MA, USA). Each condition was performed in quadruplicate. To each well 50 μl of alamarBlue cell viability reagent (Thermo Fisher Scientific, Waltham, MA, USA) was added to assess metabolic activity. Plates were incubated under microaerophilic conditions for an additional seven days. On day 14, fluorescence was measured using a BioTek Synergy H1 micromode microplate reader (Agilent, Santa Clara, CA, USA) at an excitation wavelength of 560 nm and an emission wavelength of 590 nm and presented as relative fluorescence units (RFU). Statistical analyses were conducted using Prism v. 9.5.1 (GraphPad Software). A two-way ANOVA was used to compare GFP^+^ fluorescence intensity, with Tukey’s correction for multiple comparisons. *p*-values < 0.05 were considered significant.

### *In vitro* passage to assess growth inhibition and OM disruption following incubation with IRS and ECL antibodies

To evaluate the long-term impact of antibody exposure on *TPA* growth and OM integrity, GFP^+^ treponemes were incubated with 10% heat-inactivated NRS, IRS, or ECL-specific antibodies for seven days under microaerobic conditions (passage 1 [P1]), as described above. Following incubation, treponemes were harvested and enumerated by DFM. GFP^+^ spirochetes were then normalized to 2.5 × 10^5^ organisms per well and transferred to fresh Sf1Ep monolayers in TpCM-2 medium lacking the addition of fresh antibodies. This rescue procedure was carried out over two consecutive seven-day passages (P2, days 7 through 14; P3, days 14 through 21). At the end of each passage, treponemes were harvested, enumerated by DFM to assess growth, and processed for flow cytometric analysis of OM integrity using PI, as described above. For passages P2 and P3, the input number of organisms was adjusted to account for the reduced yields observed in cultures initially treated with IRS or growth inhibiting ECL antibodies. Statistical analyses were conducted using Prism v. 9.5.1 (GraphPad Software). One-way ANOVA was used to compare spirochete counts and iMFI values across experimental groups at the end of P1, P2, and P3, with Tukey’s test applied to correct for multiple comparisons at each time point. To evaluate changes in spirochete counts and iMFI over time within individual treatment groups, two-way ANOVA with the Bonferroni correction was used. Differences were considered statistically significant at *p*-values < 0.05.

### Rabbit intradermal challenge to assess infectivity of GFP^+^*TPA* following incubation with IRS and ECL antibodies

GFP^+^*TPA* were co-cultured with Sf1Ep cells in the presence of 10% heat-inactivated NRS, IRS, or ECL-specific polyclonal antisera for seven days under microaerobic conditions as described above. On day 7, GFP^+^*TPA* were harvested and enumerated by DFM. Each condition was normalized to 1 × 10^4^ GFP^+^*TPA* in 100 μl of TpCM-2 medium and used to inoculate intradermally the shaved backs of three individual male NZW rabbits. Prior to intradermal inoculations, animals were sedated with acepromazine (1–2 mg/kg) and anesthetized with 3–5% isoflurane for the procedure. Following the injections, animals were housed in a temperature-controlled environment and examined daily to monitor lesion development. Lesion diameters were measured daily with digital calipers, starting at 10 days post-inoculation (p.i.) and continuing until sacrifice on day 31 p.i. Animals were given an overdose of a ketamine cocktail (1.5–2.0 mL containing ketamine [25–50 mg/kg], xylazine [2.5–5 mg/kg], and acepromazine [0.75–1 mg/kg]) for sedation. Once adequately sedated, pentobarbital (200 mg/kg) was injected intravenously, and a thoracotomy was performed to confirm death. Following euthanasia, cutaneous lesions were excised, thickness was measured with digital calipers and four mm punch biopsies were obtained to assess spirochete burdens by DFM and qPCR. For DFM, biopsies were placed into 150 μl of CMRL media supplemented with 10% heat-inactivated NRS and incubated at 4 °C overnight; supernatants were then examined by DFM to confirm the presence of motile spirochetes within the cutaneous skin lesions. For each sample, findings from four fields were scored in a blinded fashion as 1 (1–5 *TPA* per field), 2 (6–10 *TPA* per field), 3 (>10 *TPA* per field) and a heat map was generated using Prism (v. 9.5.1; GraphPad Software, San Diego, CA, USA). For qPCR, biopsies were placed in 500 μl of DNA/RNA Shield (Zymo Research, Tustin, CA USA) and stored at -20 °C until extraction using the DNeasy Blood and Tissue kit (Qiagen, Germantown, MD, USA). qPCR assays for *polA* and rabbit *β-actin* (Life Technologies Corporation, Carlsbad, CA, USA) were performed as previously described (23, 37). All statistical analyses were conducted using Prism v. 9.5.1 (GraphPad Software). Lesion development was analyzed using two-way ANOVA with Tukey’s correction for multiple comparisons. Lesion thickness across treatment groups was assessed using a one-way ANOVA with Bonferroni correction. Spirochete burdens were analyzed by two-way ANOVA, with Dunnett’s test applied to compare each condition to the NRS control. Differences were considered statistically significant at *p*-values < 0.05.

## Results

### FadL ECL-specific antibodies compromise outer membrane integrity during *in vitro* cultivation

**Figure 1:**
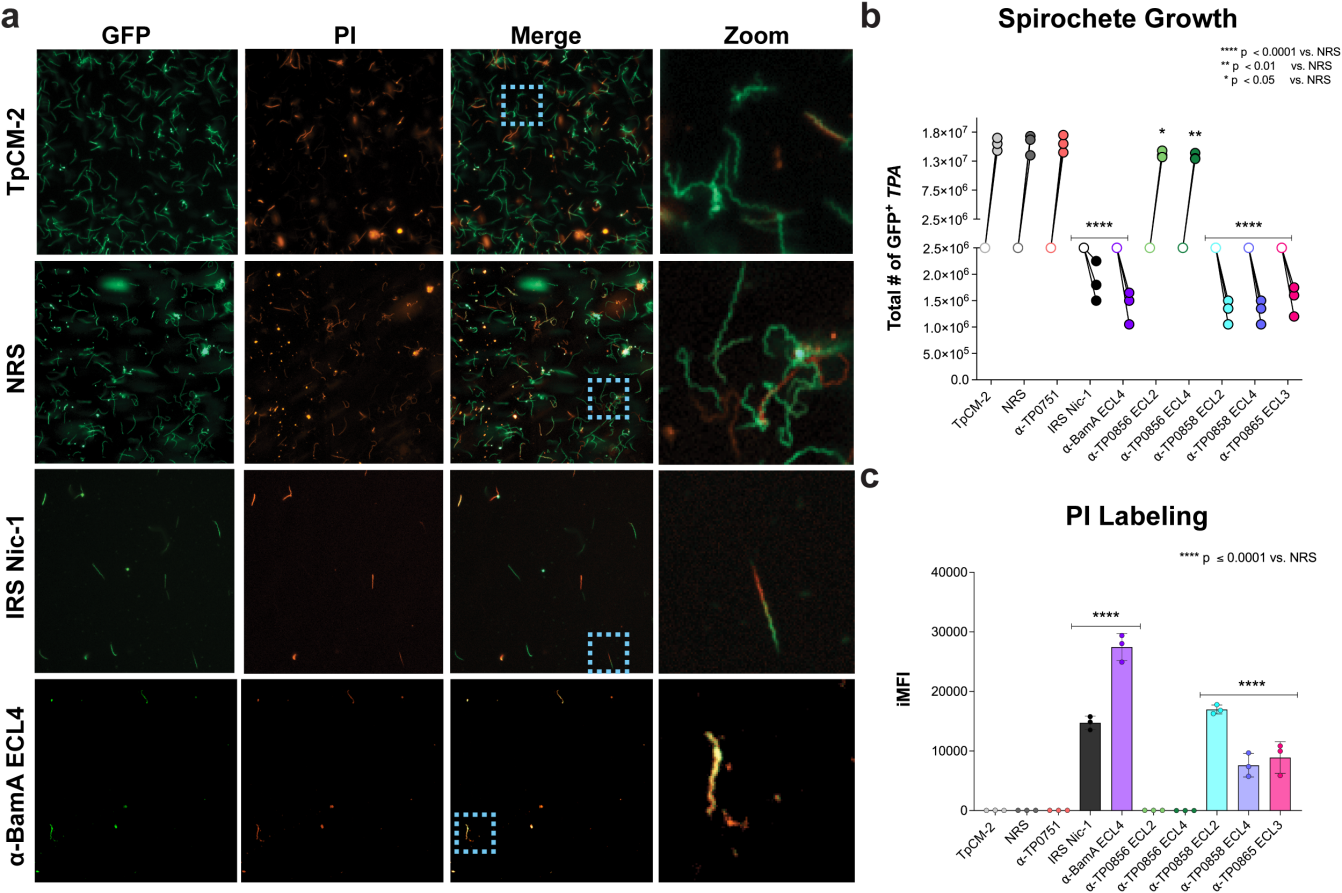
Incubation of *TPA* with IRS and ECL-specific antibodies disrupts outer membrane integrity. **(a)** Fluorescence microscopy of GFP^+^*TPA* Nichols following 7 days of co-culture with Sf1Ep cells and incubation with either TpCM-2 media alone, normal rabbit serum (NRS), serum from a Nichols-infected immune rabbit (IRS Nic-1), or α-BamA ECL4 antisera. Spirochetes were harvested using trypsin-EDTA, stained with propidium iodide (PI), and imaged in suspension. **(b)** Darkfield microscopy (DFM) enumeration of total GFP^+^*TPA* co-cultured with Sf1Ep cells in the presence of NRS, IRS, or rabbit antisera targeting either TP0751, BamA ECL4, or FadL ECLs. Open symbols represent initial spirochete densities upon seeding; closed symbols indicate densities following harvest at 7 days of co-culture. **(c)** Quantification of PI labeling by integrated mean fluorescent intensity (iMFI), calculated by multiplying the frequency of PI^+^ spirochetes by their respective MFI values. Data are presented as mean ± standard deviation (SD) from three technical replicates per condition. *p < 0.05, **p < 0.01, and ****p < 0.0001 vs. NRS. **Supplementary Table S1** contains statistical analysis of iMFI for day 7 data.

We recently demonstrated by flow cytometry that GFP-expressing *TPA* (GFP^+^*TPA*) take up the membrane impermeable dye propidium iodide (PI) during *in vitro* cultivation with heat-inactivated immune rabbit serum (IRS) and antibodies targeting ECL4 of BamA (TP0326) (11). We interpreted these results as indicating that the growth inhibitory effect of these antibodies results in disruption of the *TPA* OM. At the outset, we performed epifluorescence microscopy to substantiate this notion (**Figure 1a**). As expected, negative control conditions (TpCM-2 media alone or normal rabbit serum [NRS]) displayed a high density of GFP^+^ spirochetes, the large majority of which excluded PI. In contrast, spirochetes co-cultivated with IRS or α-BamA-ECL4 antiserum demonstrated both a reduction in overall spirochete density and a marked increase in PI^+^ staining, consistent with loss of OM integrity. Notably, in the IRS-treated condition, some spirochetes exhibited localized PI staining, with one segment of the cell PI^+^ while the remainder excluded the dye, suggesting partial OM disruption. Conversely, spirochetes incubated with α-BamA ECL4 were uniformly PI^+^.

We next sought to determine whether antibodies targeting FadL ECLs with proven growth inhibitory activity (29) similarly impact OM integrity. Consistent with previous findings (29), incubation of *TPA* with 10% α-TP0858 ECL2, α-TP0858 ECL4, or α-TP0865 ECL3 resulted in a marked reduction in spirochete numbers, while antibodies targeting ECL2 and ECL4 of TP0856 exhibited only a modest growth inhibitory effect (**Figure 1b**). Neither NRS nor antisera directed against TP0751, a periplasmic lipoprotein previously shown by our group to elicit non-opsonic, non-protective antibodies in the rabbit model (23), had any discernible effect on spirochete proliferation, whereas IRS and α-BamA ECL4 markedly inhibited growth. To quantify the frequency and extent of PI labeling across different antibody conditions by flow cytometry, we used a metric termed ‘integrated mean fluorescent intensity (iMFI)’ (44), a composite measurement that incorporates the percentage of PI^+^ spirochetes and the fluorescence intensity of PI staining. GFP^+^ spirochetes co-cultured with negative controls exhibited minimal iMFI values (44 ± 8) (**Figure 1c**, and **Supplementary Figure S2**). In contrast, spirochetes treated with IRS or α-BamA ECL4 showed significant increases in iMFI values (14,733 ± 1,121 and 27,454 ± 2,279, respectively; *p* < 0.0001 vs. NRS). Antibodies targeting TP0856 ECL2 and ECL4 yielded iMFI values (27.9 ± 19) indistinguishable from the negative controls. However, spirochetes exposed to antibodies against TP0858 ECL2 and ECL4, as well as TP0865 ECL3, displayed markedly elevated iMFI values (16,972 ± 758, 7,608 ± 1,973, and 8,908 ± 2,654, respectively; *p* < 0.0001 vs. NRS; **Supplementary Table S1**, here after **Supplementary Tables** related to statistical analyses are noted within the corresponding Figure Legends). Interestingly, among the antibodies tested, α-BamA ECL4 induced the highest iMFI (27,454 ± 2,279), followed by α-TP0858 ECL2 (16,972 ± 758), which closely mirrored the response observed with IRS (**Figure 1c**).

### Growth inhibiting ECL antibodies severely compromise spirochete metabolic activity

**Figure 2:**
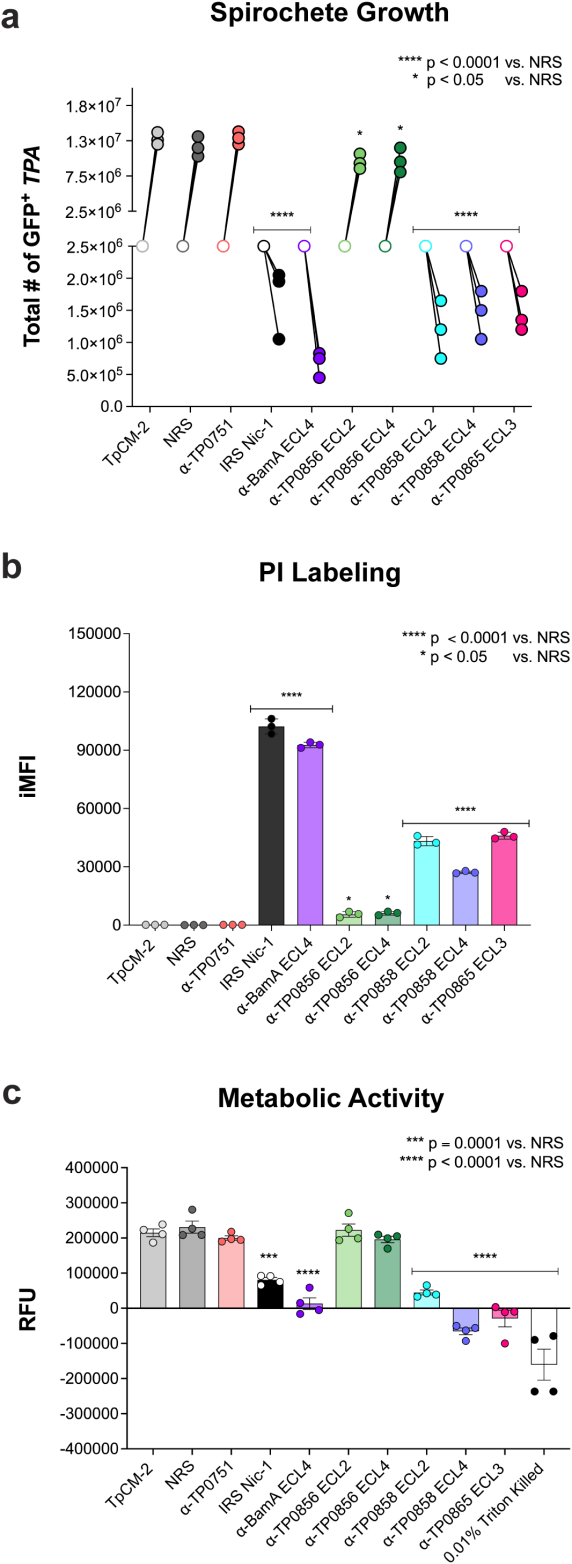
Alamar blue assay to assess the metabolic activity of *TPA* incubated with IRS and growth inhibiting ECL antibodies. **(a)** Enumeration of the total number of GFP^+^ spirochetes by DFM co-cultured with Sf1Ep cells in the presence of NRS, IRS, or antisera targeting TP0751, BamA, ECL4, or FadL ECLs. Open symbols indicate spirochete densities at seeding; closed symbols represent densities at time of harvest, 7 days post-co-culture with corresponding antisera. **(b)** Quantification of PI labeling by iMFI. Bars show means ± SD from three technical replicates. **Supplementary Table S2A** contains the statistical analysis of all conditions. **(c)** Metabolic activity of *TPA* after 7 days of co-culture was assessed using the Alamar Blue assay. Reduction of resazurin to fluorescent resorufin was quantified by relative fluorescence units (RFU). Bars represent mean ± SD from four technical replicates per condition. **Supplementary Table S2B** contains the statistical analysis of all conditions. *p < 0.05, ***p = 0.000,1 and ****p < 0.0001 vs. NRS.

As an additional measure of bacterial viability, we assessed the effects of ECL antibodies on spirochete metabolic activity using the alamarBlue assay (45). Metabolically active cells reduce the non-fluorescent blue dye resazurin to the highly fluorescent pink compound resorufin (measured as 560/590 nm excitation/emission) (45). As before, IRS and ECL-targeting antibodies (BamA ECL4, TP0858 ECL2 and ECL4, and TP0865 ECL3) with growth inhibitory activity (**Figure 2a**) resulted in a marked increase in PI iMFI values compared to the negative controls (**Figure 2b** and **Supplementary Figure S3**). We then incubated the recovered organisms from the co-cultures (normalized to 1 × 10^4^ organisms per 100 μL) with the alamarBlue reagent. As expected, negative controls, including TpCM-2 media alone, NRS and α-TP0751, exhibited strong resorufin fluorescence (**Figure 2c**) while spirochetes co-cultured with IRS or BamA ECL4 antibodies showed significantly reduced fluorescence compared to NRS-treated controls activity (*p* = 0.0001 and *p* < 0.0001, respectively; **Figure 2c**). In accord with PI staining, spirochetes treated with α-TP0856 ECL2 and ECL4 exhibited fluorescence levels comparable to NRS, while organisms incubated with antibodies targeting TP0858 ECL2 and ECL4, and TP0865 ECL3 exhibited markedly decreased resorufin fluorescence comparable to IRS and α-BamA ECL4.

### Incubation with IRS and ECL antibodies leads to sustained growth inhibition and irreparable outer membrane damage

**Figure 3:**
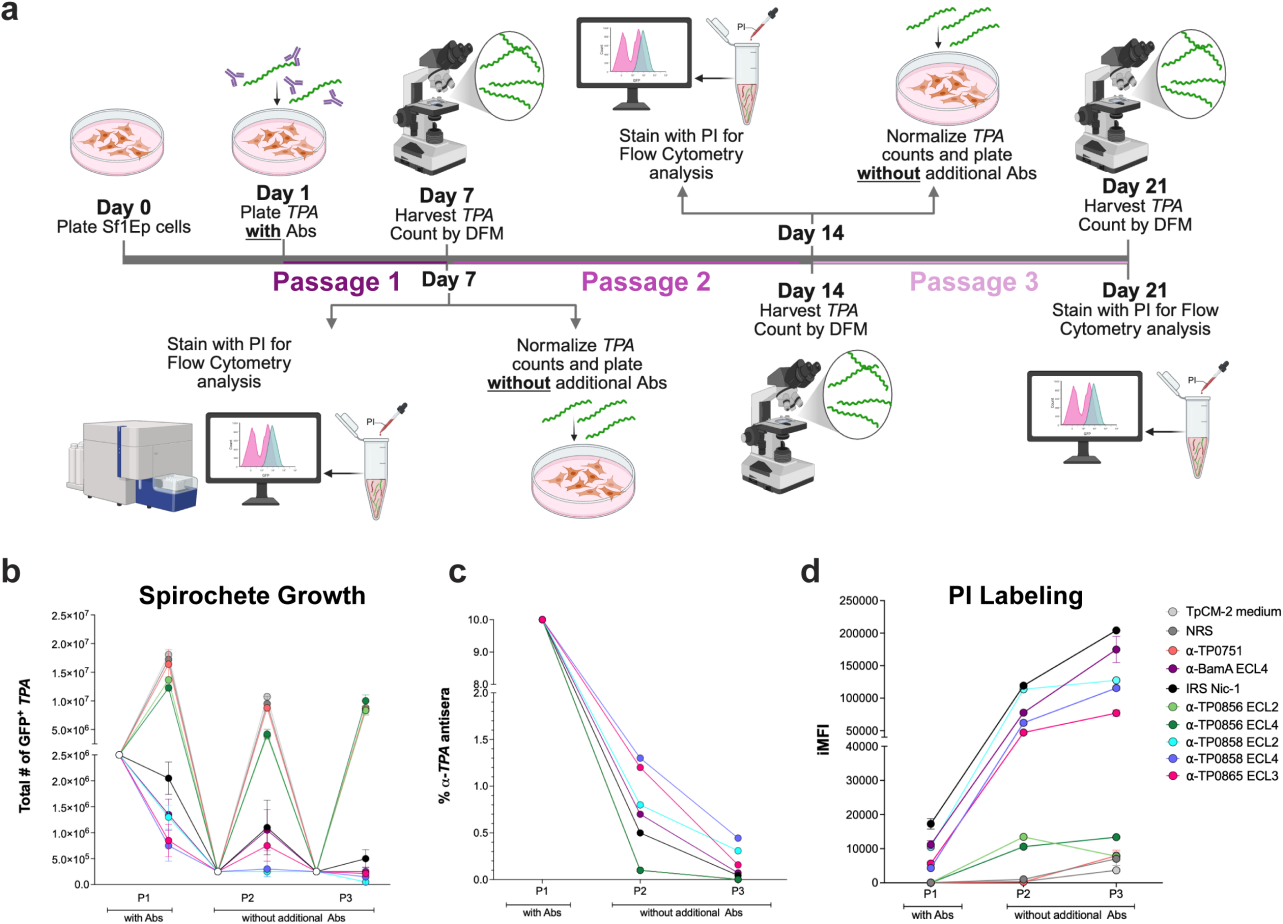
Sustained growth inhibition and progressive OM damage following incubation with IRS and ECL antibodies. **(a)** Schematic overview. Sf1Ep cells were seeded on day 0. On day 1, GFP-expressing *TPA* and 10% antibody were added. Cultures were harvested on days 7 (P1), 14 (P2), and 21 (P3), and spirochetes were quantified *via* DFM. Each harvest was split into two portions: one was stained with PI for flow cytometric analysis of OM integrity, while the other was normalized based on DFM counts and used to seed a new Sf1Ep monolayer without additional antibody. Created with BioRender.com. **(b)** Total number of GFP^+^*TPA* at the end of P1, P2 and P3 quantified by DFM. Open circles represent the initial seeding densities at the start of each passage and closed circles represent the harvested spirochete counts at the passages. Each color denotes a specific antibody condition applied during the initial 7-day incubation. All data are shown as mean ± SD from three technical replicates per condition. **(c)** Graph of the total concentration (% of total volume) of each antiserum in the individual culture passages. **(d)** PI labeling calculated by iMFI at the end of each passage. All data are shown as mean ± SD from three technical replicates per condition and **Supplementary Tables S3A-I** contains the statistical analysis of all conditions.

Prior studies have established that *TPA* populations are heterogeneous with respect to surface antibody binding (15, 20, 42, 46). Along these lines, we recently noted that *TPA* showed limited recovery following incubation with IRS and ECL-specific antibodies, suggesting the presence of antibody-resistant subpopulations (29). On the other hand, upon inspection of culture supernatants by darkfield microscopy (DFM), we also observed evidence for disruption of spirochetes incubated with growth-inhibiting antibodies, indicating a bactericidal effect. These ostensibly contradictory findings prompted us to explore in greater detail how incubation of *TPA* with IRS and ECL antibodies impacts survival and OM integrity (see workflow in **Figure 3a**). Accordingly, spirochetes initially were cultured with control sera and antisera (passage 1 [P1];10% concentration) through day seven as described above. We then re-seeded cultures on days seven (P2) and 14 (P3) with a lower than usual inoculum (2.5 × 10^5^ vs. 2.5 × 10^6^ GFP^+^*TPA* per well) to account for the reduced yields caused by IRS and growth inhibitory ECL antibodies during the initial incubation period (**Figure 3b**). The serial passages progressively dilute residual antibodies from the 10% in P1 to 1.3%-0.1% in P2 and 0.44% - 0.001% P3 (**Figure 3c**).

Negative controls showed robust growth during P2 with extremely low iMFI values unchanged from P1 (**Figure 3b, d** and **Supplementary Figure S4**), while organisms incubated with IRS, α-BamA ECL4, and α-TP0865 ECL3 recovered modestly but, unexpectedly, with iMFI values markedly above their post-P1 levels (**Figure 3b, d** and **Supplementary Figure S4**). Spirochetes incubated with α-TP0858 ECLs showed no recovery in growth during P2 and, like their IRS- and α-BamA ECL4-incubated counterparts, dramatically elevated iMFI values. Spirochetes incubated with α-TP0856 ECLs displayed small, albeit significant, growth inhibition in P2, as in P1, but with substantial increases in iMFI levels (**Figures 3b, d**). At the conclusion of P3, cultures previously incubated with IRS, α-BamA ECL4, or α-TP0865 ECL3 showed negligible increases in spirochete numbers along with iMFI values even greater than those at the end of P2. Organisms previously incubated with α-TP0858 ECLs also showed no improvement in numbers at the conclusion of P3 with iMFI values equal to (ECL2) or greater (ECL4) than at the end of P2. Cultures incubated with α-TP0856 ECLs showed full growth recovery during P3 but with sustained, elevated iMFI values.

### IRS and ECL growth inhibitory antibodies neutralize *TPA* infectivity

**Figure 4:**
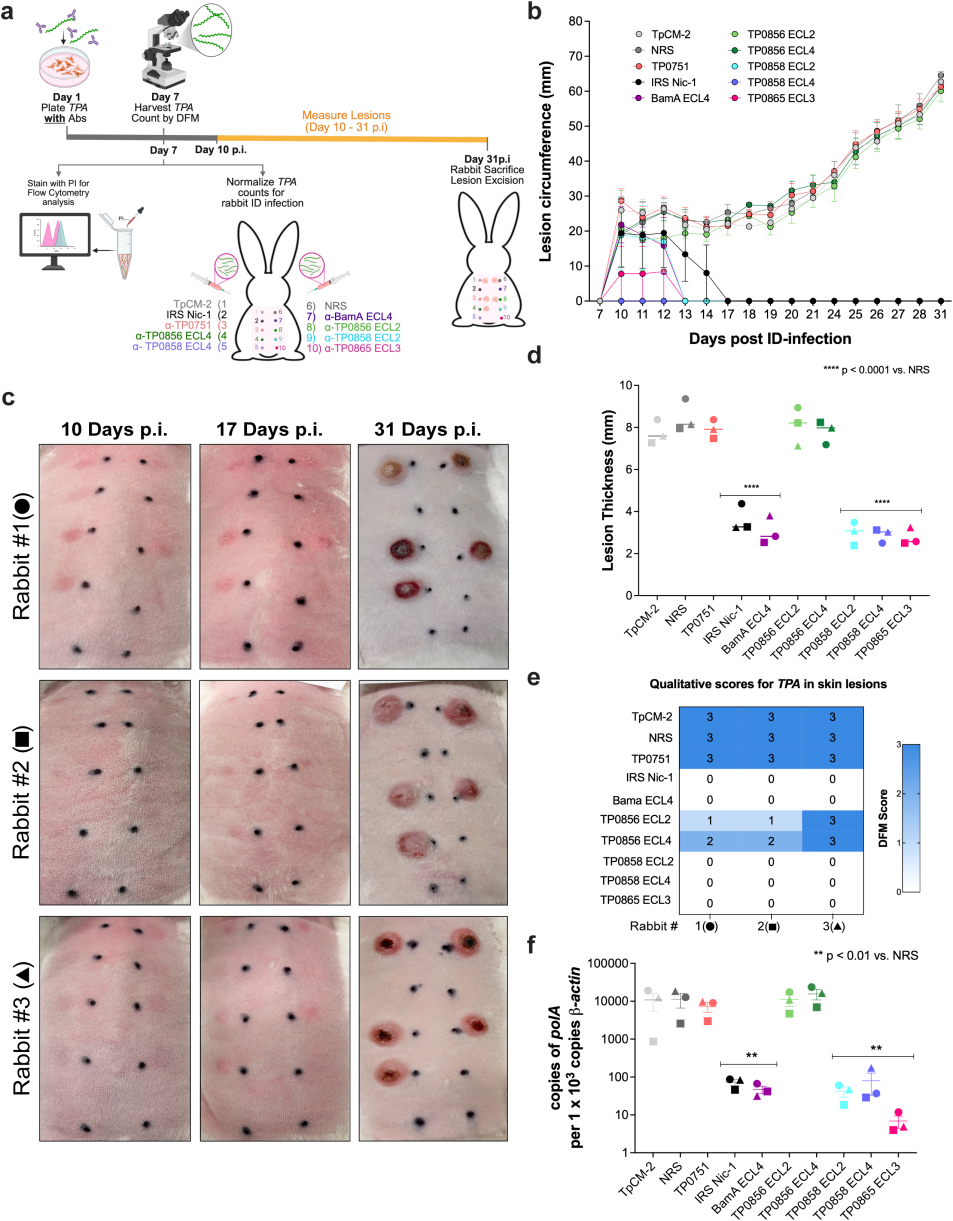
Assessment of *TPA* infectivity by intradermal challenge of rabbits. **(a)** Schematic overview. GFP^+^*TPA* were co-cultured with antibodies and harvested at day 7 for quantification via DFM. Harvested samples were divided into two groups: one was normalized based on DFM counts and used for intradermal challenge of rabbits (n = 3). Each rabbit received one injection site per condition on its shaved back, with conditions replicated across all rabbits. The other group was stained with PI for flow cytometric analysis of membrane integrity (see **Supplementary Figure S5**). Lesion development was monitored daily from the onset of visible lesions at control sites on day 7 through sacrifice at day 31 post-infection (p.i.). Following sacrifice, lesions were excised to measure overall lesion thickness and quantification of *TPA* burdens by DFM and qPCR. Created with BioRender.com. **(b)** Lesion circumferences (mm). Measurements were taken from the onset of lesion appearance, starting at day 7 p.i. through sacrifice on day 31 p.i. Values represent mean ± SD. **(c)** Representative photographs of the shaved backs of all three rabbits showing lesion development at days 10, 17, and 31 p.i. Each rabbit is identified by a different symbol: circle (rabbit 1), square (rabbit 2), and triangle (rabbit 3). **(d)** Lesion thickness (mm, means ± SD) measured at the time of sacrifice. ****p < 0.0001 vs. NRS. **(e)** Heatmap of *TPA* density in skin lesions determined by DFM. Spirochete burden was qualitatively scored based on visual density: 0 = no detectable spirochetes; 1 = 1–5 *TPA* per field; 2 = (6–10 *TPA* per field); and 3 = (>10 *TPA* per field). **(f)** Treponemal burdens quantified by *polA* qPCR normalized to 10^3^ copies of rabbit *β-actin*. Each data point corresponds to lesion burdens from individual animals (rabbit 1: circle; rabbit 2: square; rabbit 3: triangle). Data represents mean ± SD from three animals. **Supplementary Table S4** contains the statistical analysis of all conditions. **p < 0.01 vs. NRS.

While the above results show that spirochetes incubated with growth inhibitory antibodies have negligible capacity for recovery with sequential passage during *in vitro* cultivation conditions, the possibility remained that they retain the ability to cause lesions upon intradermal inoculation into infection-naïve rabbits. Per the workflow in **Figure 4a**, spirochetes co-cultured for seven days under the conditions used above were harvested, enumerated by DFM, and inoculated intradermally into the shaved backs of rabbits (n = 3) at 1 × 10^4^ organisms per site. In parallel, samples from the cultures were enumerated, stained with PI, and analyzed by flow cytometry confirmed that the *in vitro* results mirrored those in the preceding experiments (**Supplementary Figure S5** vs. **Figures 1**–**3**).

Sites inoculated with spirochetes incubated with α-TP0856 ECL2 or ECL4 developed lesions with a time course to ulceration indistinguishable from negative controls. In contrast, spirochetes incubated with α-BamA ECL4 and IRS developed erythematous lesions by day 10 post-infection (p.i.) that resolved by days 13 and 17, respectively (**Figures 4b, c**). Lesion development for spirochetes incubated with α-TP0858 ECL2 and α-TP0865 ECL3 mirrored that of α-BamA ECL4, while no lesions were observed at any time point with α-TP0858 ECL4-treated spirochetes. Lesions were excised on day 31 p.i. (**Figure 4c**) for measurement of thickness (**Figure 4d**) and assessment of spirochete burdens by DFM and *polA* qPCR (**Figures 4e, f**, respectively). Sites injected with spirochetes incubated with α-TP0856 ECLs 2 and 4 were comparable in thickness to negative controls while those from organisms incubated with IRS, α-BamA ECL4, α-TP0858 ECL2, α-TP0858 ECL4, and α-TP0865 ECL3 were markedly thinner. DFM confirmed the presence of motile spirochetes in lesions from the negative control groups. No spirochetes were recovered from sites inoculated with IRS, α-BamA ECL4, α-TP0858 ECL2, α-TP0858 ECL4, and α-TP0865 ECL3 treated spirochetes. Lesions from sites inoculated with organisms incubated with α-TP0856 ECLs 2 and 4 contained live spirochetes, albeit in lower numbers than the negative controls. Consistent with the DFM findings, *polA* qPCR measured high copy numbers in lesions from negative control groups and significantly reduced burdens in lesions from organisms incubated with IRS, α-BamA ECL4, α-TP0858 ECL2, α-TP0858 ECL4, and α-TP0865 ECL3. In contrast, lesions from α-TP0856 ECLs 2 and 4-treated groups contained *polA* copy numbers comparable to the negative controls.

## Discussion

Clarifying the role of antibodies in spirochete clearance and protective immunity has long been a central objective of syphilis research. Obtaining convincing evidence for antibody-mediated clearance mechanisms in humans has proven difficult, as the disease persists, and even progresses, in the face of a robust humoral response. The experimental rabbit model, on the other hand, has provided compelling evidence that antibodies can be protective, with passive immunization using IRS shown to suppress lesion formation following intradermal challenge (13, 16). Subsequent groundbreaking *ex vivo* studies by Lukehart and Miller (15) showing that IRS contains opsonic antibodies established a presumptive mechanistic basis for antibody-mediated *TPA* clearance along with the now widely held notion that the presence of opsonic antibodies can be considered a hallmark of protective immunity. Advances in defining the molecular architecture of the *TPA* cell envelope have since narrowed the likely targets of functional antibodies to ECLs of OMPs (21). Recent work using the *in vitro* cultivation system has provided definitive evidence that antibodies directed against specific ECLs can markedly impair *TPA* viability (11, 29). Furthermore, IRS contains antibodies against BamA ECL4, FadL ECLs, and likely other ECLs (29, 31), supporting the assumption that loop-specific antibodies elicited by *TPA* infection mediate its protective effects. As a whole, these findings guide a structure-based approach to investigate the physiological roles of *TPA* OMPs in the service of rational vaccine design while enabling the development of *in vitro* assays with the potential to predict protective capacity *in vivo*.

Vaccine development for syphilis has moved away from expression of full-length OMPs to scaffolds displaying ECLs (29, 31), resulting in the need for robust methodologies to evaluate and compare the vaccine potential of individual ECLs. We recently developed a GFP-expressing strain of *TPA* that laid the groundwork for a flow cytometric assay to quantitate OM damage inflicted by ECL antibodies in IRS and ECL-specific antibodies elicited by immunization (11). This method is based on our finding that functional antibodies (*i*.*e*., IRS and α-BamA ECL4) allow uptake of the membrane-impermeant dye PI, whereas spirochetes incubated with control antibodies excluded it. To stain organisms, PI also must breach the cytoplasmic membrane to bind DNA; thus, antibodies that result in PI staining eventually result in disruption of the entire cell envelope. Fluorescence microscopy provided visual evidence for double labeling (GFP^+^/PI^+^) of bacterial cells. With this assay, herein, we showed that antibodies to ECLs of two FadL proteins, TP0858 and TP0865, that prevented *in vitro* growth also resulted in PI staining. Assessment of metabolic activity correlated closely with PI staining, providing additional evidence that the antibody-mediated breaches in the OM seriously impaired spirochete viability. Notably, antibodies to individual ECLs resulted in levels of PI staining comparable to that of IRS. This result points to the greater potency of ECLs generated by immunization and suggests that growth inhibition by IRS is a “multi-ECL hit” phenomenon, whereby antibodies likely target multiple ECLs to collectively reproduce the inhibitory effect observed with individual ECL-specific antisera.

In *E. coli*, antibodies to BamA ECL4 trap the protein in its ‘open’ conformation, blocking OM biogenesis (47). Antibodies targeting ECL4 of *TPA* BamA presumably exert an analogous effect. One would expect this functional lesion to have a direct effect on OM permeability as well as causing downstream physiologic perturbations that lead to PI staining (**Supplementary Figure S1B**). However, this proposed mechanism for antibodies against BamA ECL4 cannot apply to FadL (*e.g.*, TP0858) ECL antibodies. Instead, it seems plausible that anti-FadL ECLs interfere with FadL import functions, “starving” the bacterium of essential nutrients, that likely include flavin cofactors and fatty acids required for production of lipoproteins and phospholipids (**Supplementary Figure S1B**). The result is a combined metabolic/structural lesion that impairs membrane integrity and viability. Nutritional immunity is traditionally defined as host defense mechanisms that starve bacterial pathogens of transition metals (48). In recent years, the concept has been extended to “metabolic immunity” - restriction of diverse nutrients required to maintain metabolic homeostasis (49). Production of FadL ECL antibodies during syphilitic infection points to a novel type of metabolic immunity based on adaptive immune responses that functionally impair OM transporters. AlphaFold 3 (50) models *TPA* FadLs with unique structural features that distinguish them from Gram-negative FadL fatty acid importers (51); these differences suggest novel pathways for extracting hydrophobic molecules from host cells and body fluids and shuttling them across the *TPA* OM (**Supplementary Figure S1C**). What accounts for the strikingly different results for TP0856 ECL antibodies in the *in vitro* system? The inability of TP0856 ECL antibodies to find their targets on the *TPA* surface cannot be the explanation, given that TP0856 is well expressed *in vitro* and that antibodies to ECL2 and ECL4 of TP0856 are strongly opsonic (29). The most plausible explanation is that TP0856 is functionally redundant with other FadLs. This explanation is supported by our recent discovery of circulating *TPA* strains with truncated TP0856s (51).

*TPA* populations display heterogeneity in surface antibody binding with IRS whether assessed by opsonophagocytosis (15, 42) or by immunolabeling (20, 46). Heterogeneity also has been observed by immunolabeling with antibodies to individual OMPs (27, 52). In our *in vitro* cultivation system, we previously saw evidence for survival of a subpopulation of organisms as well as unmistakable evidence for a lytic, bactericidal effect (29). Efforts to understand this dichotomy seemed warranted given its obvious implications for evasion of protective antibodies following immunization. Due to the fragile nature of the spirochete’s OM (10, 53), removal of anti-ECL antibodies by centrifugation prior to passage was not an attractive strategy; instead, we used serial passage in the presence of decreasing levels of ECL antibodies to assess OM integrity and the capacity of growth-inhibited organisms to recover. During P2, we observed modest recovery of organisms incubated with IRS and two growth-inhibiting ECL antibodies (α-TP0865 ECL3 and α-BamA ECL4) not sustained during P3; PI staining indicated that these antibodies also induced irreparable, progressive damage to the spirochete population. TP0858 ECL antibodies showed the greatest potency; spirochetes incubated with the TP0858 antibodies during P1 showed no recovery along with marked uptake of PI during both subsequent passages. The results with TP0856 ECL antibodies were intriguing and not easily explainable. As in previous experiments (29), TP0856 ECL antibodies had little discernible effect on growth *in vitro* or PI staining during the initial culture period. However, the serial passage data indicated that the TP0856 ECL antibodies exerted a subtle effect on the fitness and physiology of the organisms from which they could recover, though with sustained alterations in membrane permeability detected by PI staining. Regardless of cause, this result sounds a cautionary note about equating increased permeability to PI with diminished cell viability, and it accords with studies with other bacteria showing that under various conditions PI staining can underestimate cell viability (54, 55).

The serial passage experiments revealed that a subpopulation of organisms could survive the initial incubation with IRS and some ECL antisera, although their capacity for recovery *in vitro* was extremely poor. We next asked if it was possible for these survivors to recover and cause lesions if inoculated intradermally into naïve rabbits, which are exquisitely susceptible to *TPA* infection. The rabbit model of syphilis provides an established and reproducible system for testing the biological relevance of these *in vitro* observations. Although the model does not fully recapitulate the progression of human disease (*e*.*g*., lack of disseminated lesions or progression to late-stage complications, including neurological involvement) (56), its close correspondence to human infection in lesion evolution, histopathology, and immune response makes it particularly well suited for evaluating antibody-mediated effects on infectivity (57). Using a modified version of the *in vitro*–*in vivo* neutralization test developed by Bishop and Miller (13) — which differs from their complement-dependent assay by employing a much longer incubation period with ECL antibodies in the absence of complement — we found that our *in vitro* results were an accurate gauge of infectivity. During the first 10 days, sites inoculated with spirochetes incubated with growth inhibiting antibodies developed erythematous lesions similar to controls. However, the lesions resolved between days 13 to 17, while control lesions progressed to ulceration. Although regions inoculated with *TPA* pre-incubated with growth-inhibiting antibodies were darkfield negative at the time of excision, the qPCR signal detected at resolved sites likely reflected residual *TPA* DNA from the inocula. While transient lesions might have represented an inflammatory response to the inoculum, the absence of lesions with one ECL antiserum (TP0858 ECL4) suggests they were produced by surviving spirochetes. Notably, despite similar *in vitro* activity, transient lesions occurred at sites inoculated with spirochetes exposed to TP0858 ECL2 but not ECL4 antibodies, suggesting subtle functional differences between targets on the same OMP. Consistent with the *in vitro* data, spirochetes treated with TP0856 ECL antibodies showed only slight reductions in infectivity based on DFM counts, with no significant differences in lesion development or bacterial burden measured by qPCR. Together, these results highlight the predictive power of *in vitro* assays for *in vivo* infectivity and uncover subtle but likely meaningful functional differences among ECL-targeting antibodies.

A correlate of protection, broadly defined as an immune marker predictive of protection against a specified clinical disease endpoint, is widely considered an essential requirement for vaccine studies (32–36). Ideally, a correlate of protection should be based on a known mechanism of protection. While typically thought of in the context of clinical trials, correlates of protection are equally relevant to animal models. In experimental syphilis, there is a long history of efforts to define antibody-based correlates of protection beginning with the *TPA* immobilization (TPI) test (58, 59). However, investigators in the pre-molecular era disagreed about whether TPI titers reflected protective immunity (60–62). Bishop and Miller maintained that titers in their *in vitro*–*in vivo* neutralization assay correlated with protection following intratesticular inoculation (63). As noted earlier, in recent years, opsonophagocytosis has been considered the primary mechanism for spirochete clearance (14, 15, 37–39), but results from our *in vitro* killing assay suggest this concept warrants re-evaluation. The dynamics of macrophage uptake *in vitro* differ substantially from *in vivo* conditions, where motile spirochetes can evade phagocytosis by ‘outrunning’ much slower macrophages. An alternative scenario, which we favor, is that antibodies directly injure the bacteria with or without complement, while the primary role of macrophages is to remove debilitated spirochetes or spirochete remnants. As in the Bishop and Miller studies, incubation of spirochetes with IRS neutralized their infectivity (63). This contrasts with what was observed when rabbits were passively immunized with IRS (13, 64); in the latter circumstance, spirochete infectivity was suppressed but not eliminated. Although Bishop and Miller attributed this survival phenomenon to spirochetes residing in intracellular niches, our *in vivo* microscopy studies with GFP-expressing *TPA* argue against this idea (11). In any event, the critical question then becomes whether ECL antibodies that are cidal *in vitro* can find their targets *in vivo* and exert comparable neutralizing effects. Experiments in which immunized animals with strongly bactericidal ECL antibodies are challenged should provide the answer.

## Data Availability

The original contributions presented in the study are included in the article/**Supplementary Material**. Further inquiries can be directed to the corresponding author.
