# Supplementary material for: Antibodies directed against extracellular loops of FadL orthologs disrupt outer membrane integrity and neutralize infectivity of Treponema pallidum, the syphilis spirochete
Source: Front Immunol. 2026 Jan 2;16:1724458. doi: 10.3389/fimmu.2025.1724458 (PMC12808356; doi:10.3389/fimmu.2025.1724458)
Supplement: Supplementary file 1 [file DataSheet1.pdf]

# SUPPLEMENTARY FIGURES

## Antibodies directed against extracellular loops of FadL orthologs disrupt outer membrane integrity and neutralize infectivity of *Treponema pallidum*, the syphilis spirochete

Kristina N. Delgado[1,2], Crystal F. Vicente[1], Carson J. La Vake[1], Everton Bettin[2], Melissa J. Caimano[1,2,3,4], Justin D. Radolf[1,2,3,4,5,6], and Kelly L. Hawley[1,2,4,5,7*]

[1]Department of Pediatrics, UConn Health, Farmington, CT, United States.

[2]Department of Medicine, UConn Health, Farmington, CT, United States.

[3]Department of Molecular Biology and Biophysics, UConn Health, Farmington, CT, United States.

[4]Department of Research, Connecticut Children's Research Institute, Hartford, CT, United States

[5]Department of Immunology, UConn Health, Farmington, CT, United States.

[6]Department of Genetics and Genome Sciences, UConn Health, Farmington, CT, United States.

[7]Division of Infectious Diseases and Immunology, Connecticut Children's, Hartford, CT, United States.

**Corresponding author**: Kelly L. Hawley, PhD

263 Farmington Avenue

Farmington, CT 06030

Tel: (860) 679-2087

hawley@uchc.edu

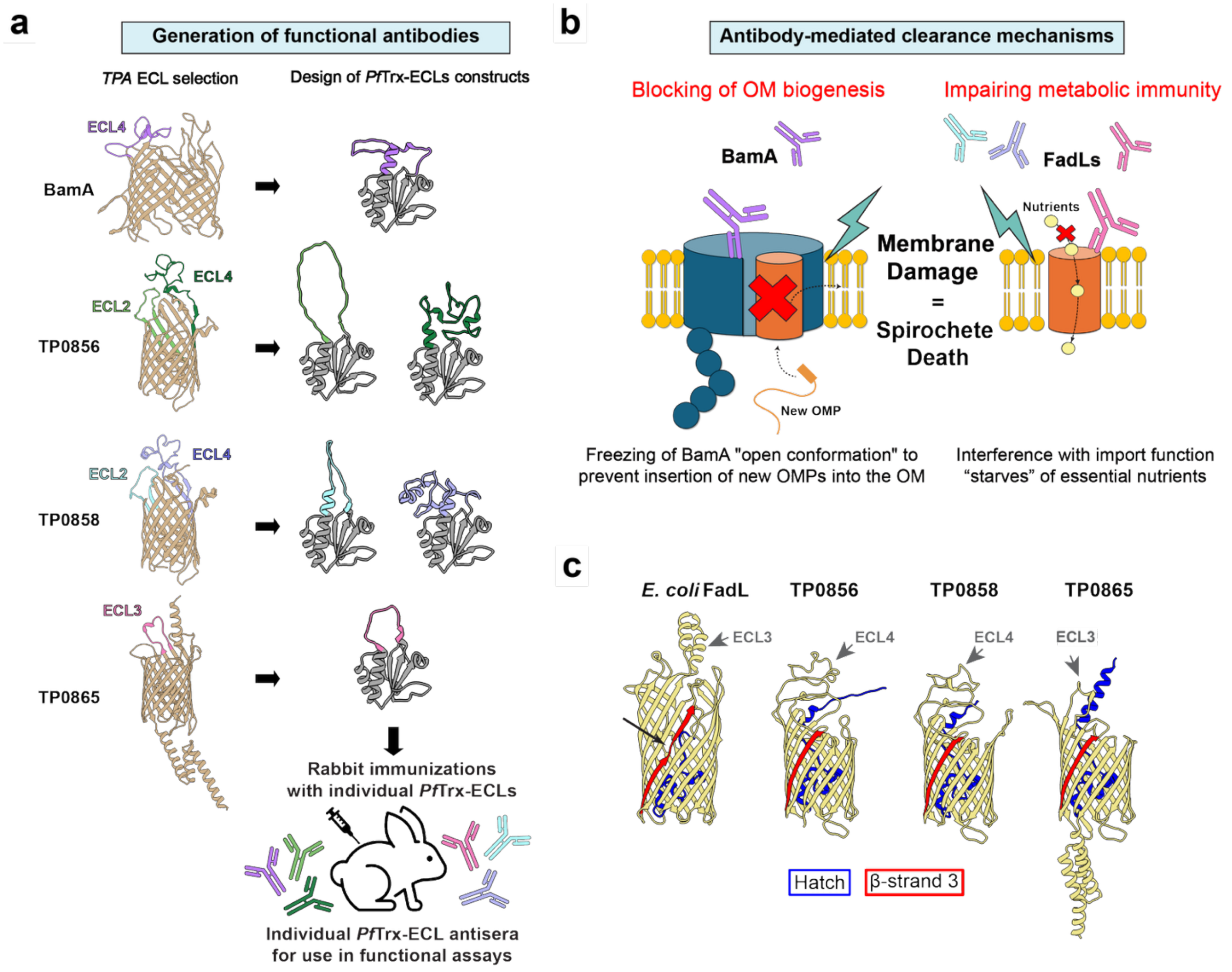


**Figure S1 | Schematic of *Pf*Trx-scaffolded *TPA* ECL immunization and proposed antibody-mediated killing mechanisms. (A)** Six highly antigenic ECLs were selected from BamA and the FadL orthologs TP0856, TP0858, and TP0865, as colored/highlighted in the respective AlphaFold 3 models (left). Each selected ECL was cloned into a *Pyrococcus furiosus* thioredoxin (*Pf*Trx) scaffold (right) and used as immunogens in rabbits. Antisera raised against individual *Pf*Trx–ECLs were subsequently evaluated for functional activity. **(B)** Proposed mechanisms of antibody-mediated clearance of *TPA*. ECL-targeting antibodies may (left) block BamA-dependent outer membrane (OM) biogenesis by stabilizing BamA in an "open conformation", thereby preventing insertion of new OMPs into the OM, or (right) impair metabolic homeostasis by interfering with FadL-mediated import of essential nutrients, effectively "starving" the organism. **(C)** Ribbon

models highlight unique structural features that distinguish the *E. coli* FadL fatty acid importer (PDB: 1T1L) from the *TPA* FadL orthologs TP0856, TP0858, and TP0865 (AlphaFold 3-predicted). The *E. coli* FadL structure contains a distinct “kink” (black arrow) in β-strand 3 (red ribbon), which is absent in all *TPA* FadLs. The hatch domain (blue ribbon), which occludes the β-barrel lumen to regulate substrate passage, is predicted to extend into the extracellular space in the *TPA* FadLs. Grey arrows indicate the longest ECL in each OMP.

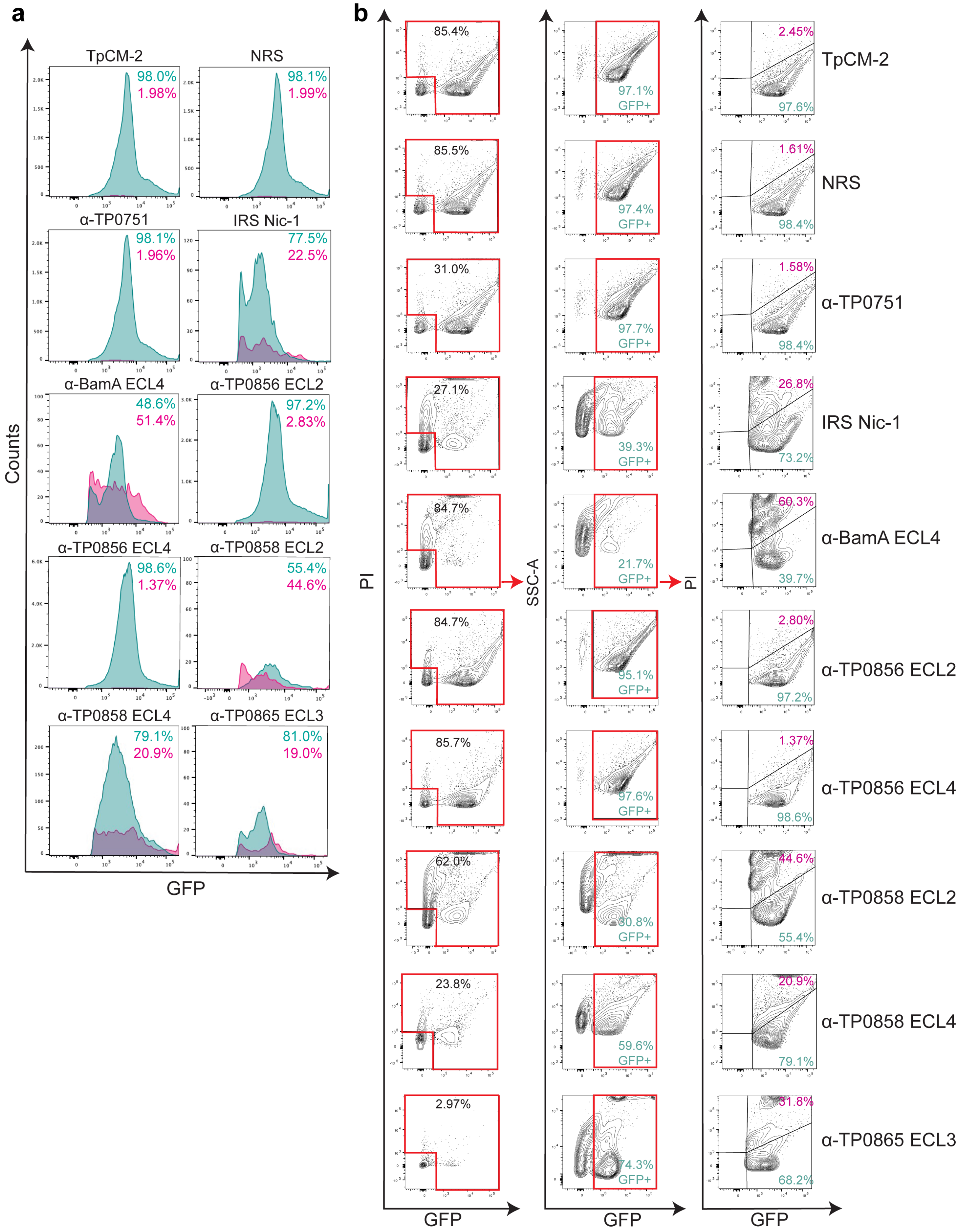

a
TpCM-2
NRS
α-TP0751
IRS Nic-1
α-BamA ECL4
α-TP0856 ECL2
α-TP0856 ECL4
α-TP0858 ECL2
α-TP0858 ECL4
α-TP0865 ECL3
98.0%
1.98%
98.1%
1.99%
98.1%
1.96%
77.5%
22.5%
48.6%
51.4%
97.2%
2.83%
98.6%
1.37%
55.4%
44.6%
79.1%
20.9%
81.0%
19.0%
Counts
GFP
b
PI
SSC-A
PI
GFP
GFP
GFP
85.4%
85.5%
31.0%
27.1%
84.7%
84.7%
85.7%
62.0%
23.8%
2.97%
97.1% GFP+
97.4% GFP+
97.7% GFP+
39.3% GFP+
21.7% GFP+
95.1% GFP+
97.6% GFP+
30.8% GFP+
59.6% GFP+
74.3% GFP+
2.45%
97.6%
1.61%
98.4%
1.58%
98.4%
26.8%
73.2%
60.3%
39.7%
2.80%
97.2%
1.37%
98.6%
44.6%
55.4%
20.9%
79.1%
31.8%
68.2%

**Figure S2 | Flow cytometric analysis of PI-labeled GFP$^+$ *TPA* for assessing outer membrane integrity. (A)** Representative flow cytometry histograms showing the proportion of GFP$^+$ *TPA* that are PI$^-$ (cyan) or PI$^+$ (magenta) after 7-day incubation with the indicated sera (10% final concentration). **(B)** Gating strategy used to assess OM disruption of *in vitro*-cultivated GFP$^+$ *TPA.* Flow cytometric panels were used to exclude non-spirochetal (*i.e.*, double negative) events and then to assess the percentage of PI$^+$ organisms within the GFP$^+$ population for each serum condition. Results are representative of three technical replicates.

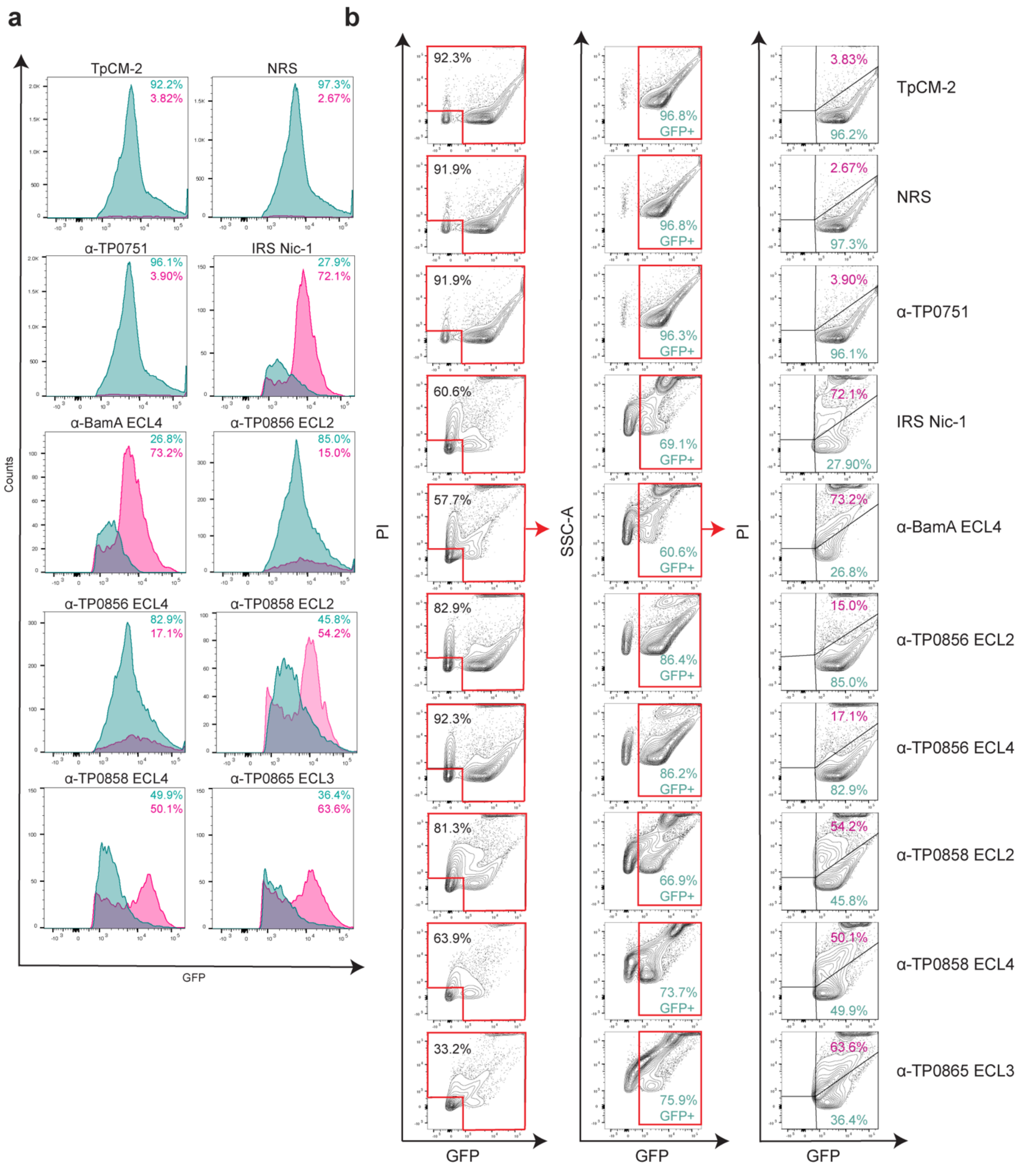


**Figure S3 | Flow cytometric analysis of *TPA* outer membrane integrity. (A)** Representative flow cytometry histograms showing the proportion of GFP$^+$ *TPA* that are PI$^-$ (cyan) or PI$^+$ (magenta) after 7-day incubation with the indicated sera (10% final concentration). **(B)** Gating strategy used to assess OM disruption of *in vitro*-cultivated GFP$^+$ *TPA*.

**a**

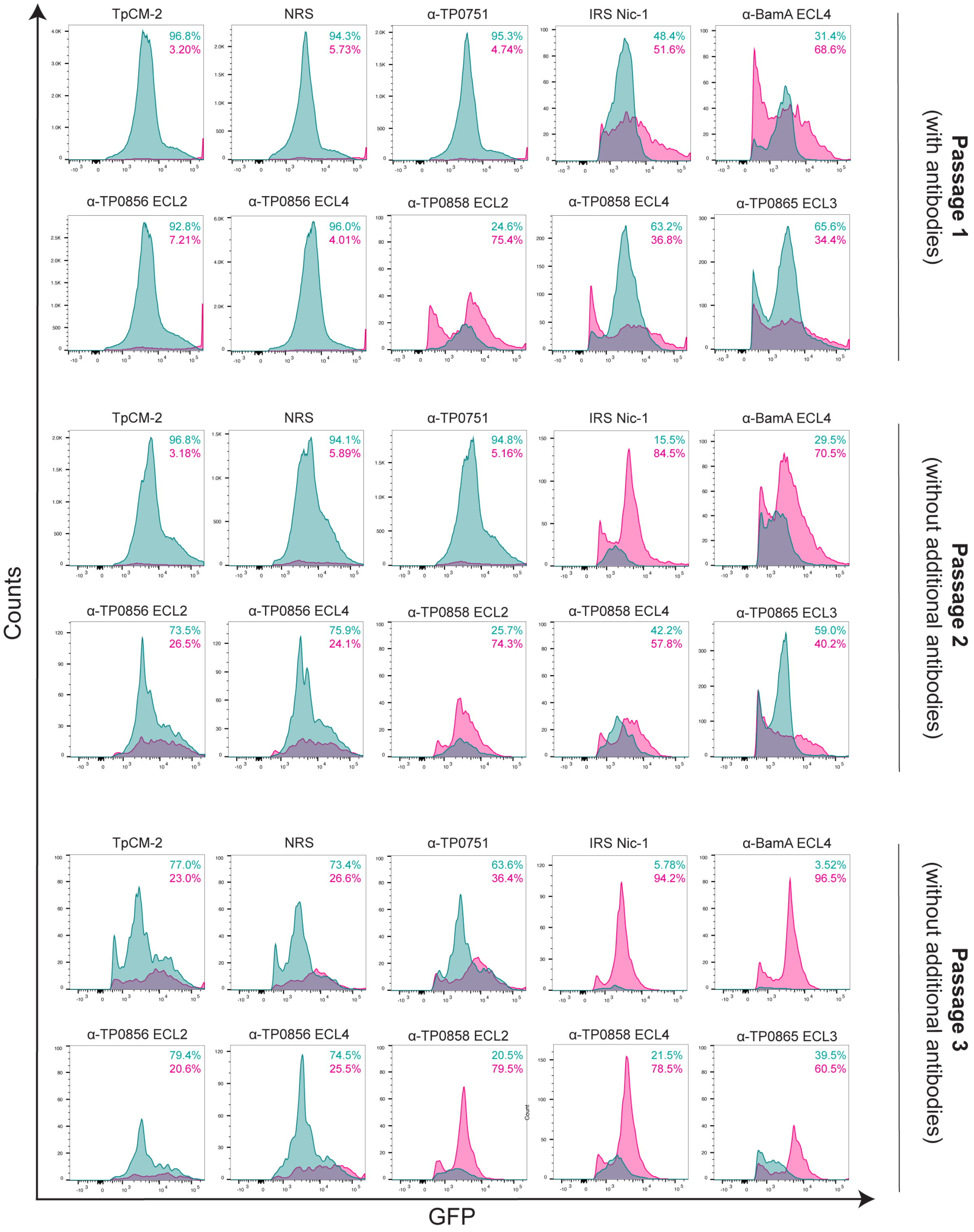
Counts
GFP
Passage 1
(with antibodies)
TpCM-2
96.8%
3.20%
NRS
94.3%
5.73%
α-TP0751
95.3%
4.74%
IRS Nic-1
48.4%
51.6%
α-BamA ECL4
31.4%
68.6%
α-TP0856 ECL2
92.8%
7.21%
α-TP0856 ECL4
96.0%
4.01%
α-TP0858 ECL2
24.6%
75.4%
α-TP0858 ECL4
63.2%
36.8%
α-TP0865 ECL3
65.6%
34.4%
Passage 2
(without additional antibodies)
TpCM-2
96.8%
3.18%
NRS
94.1%
5.89%
α-TP0751
94.8%
5.16%
IRS Nic-1
15.5%
84.5%
α-BamA ECL4
29.5%
70.5%
α-TP0856 ECL2
73.5%
26.5%
α-TP0856 ECL4
75.9%
24.1%
α-TP0858 ECL2
25.7%
74.3%
α-TP0858 ECL4
42.2%
57.8%
α-TP0865 ECL3
59.0%
40.2%
Passage 3
(without additional antibodies)
TpCM-2
77.0%
23.0%
NRS
73.4%
26.6%
α-TP0751
63.6%
36.4%
IRS Nic-1
5.78%
94.2%
α-BamA ECL4
3.52%
96.5%
α-TP0856 ECL2
79.4%
20.6%
α-TP0856 ECL4
74.5%
25.5%
α-TP0858 ECL2
20.5%
79.5%
α-TP0858 ECL4
21.5%
78.5%
α-TP0865 ECL3
39.5%
60.5%

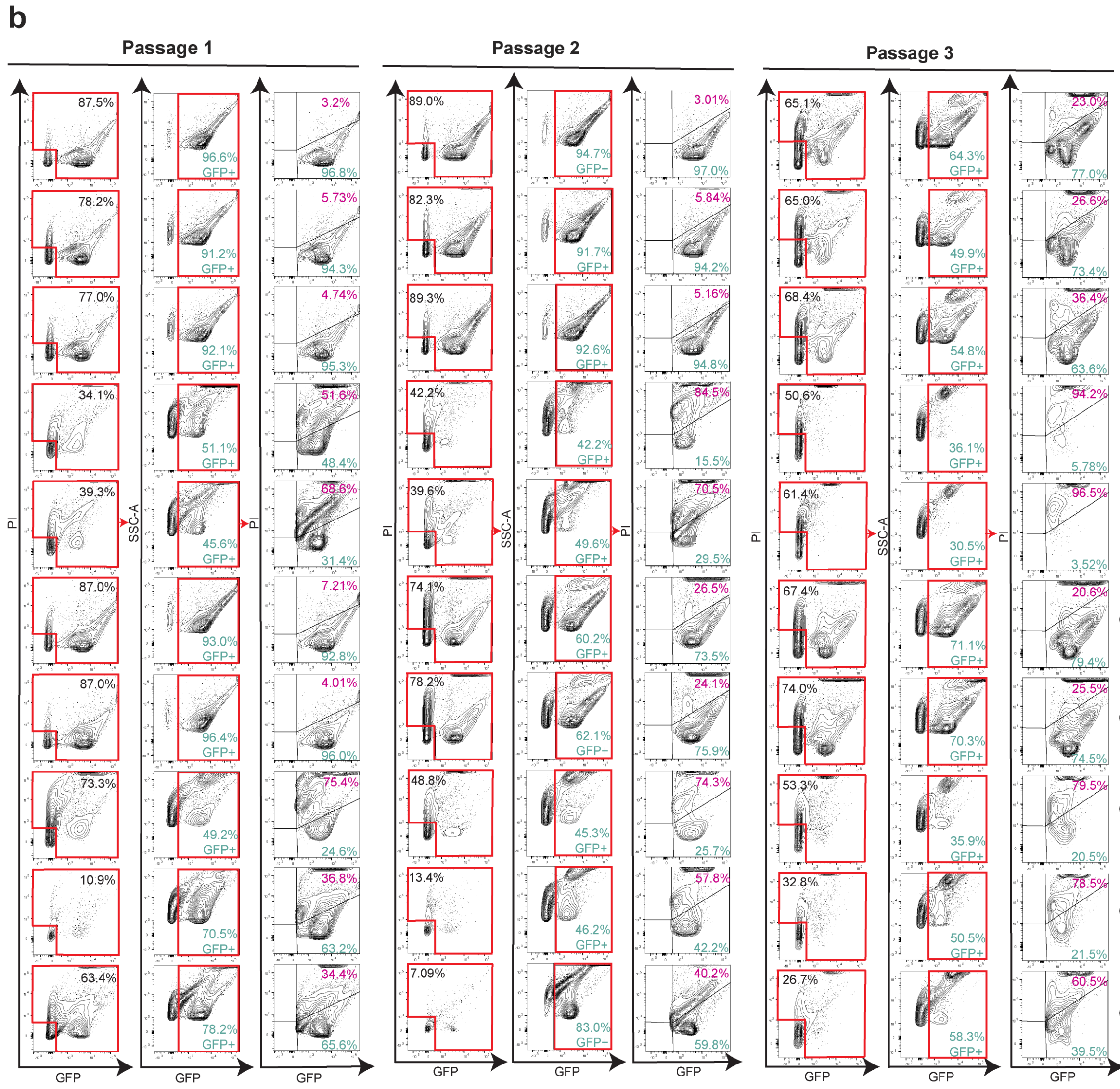


**Figure S4 | Flow cytometric analysis of *TPA* OM integrity following serial passage. (A)** Representative flow cytometry histograms showing the proportion of GFP$^{+}$ *TPA* that are PI$^{-}$ (cyan) or PI$^{+}$ (magenta) following 7-day incubation (Passage 1) with the indicated sera (10% final concentration), and after two subsequent 7-day passages in antibody-free medium, day 14 (Passage 2) and day 21 (Passage 3). **(B)** Gating strategy used to assess OM disruption of *in vitro*-cultivated GFP$^{+}$ *TPA* at the end of Passage 1 (with antibodies) and Passages 2 and 3 (without additional antibodies).

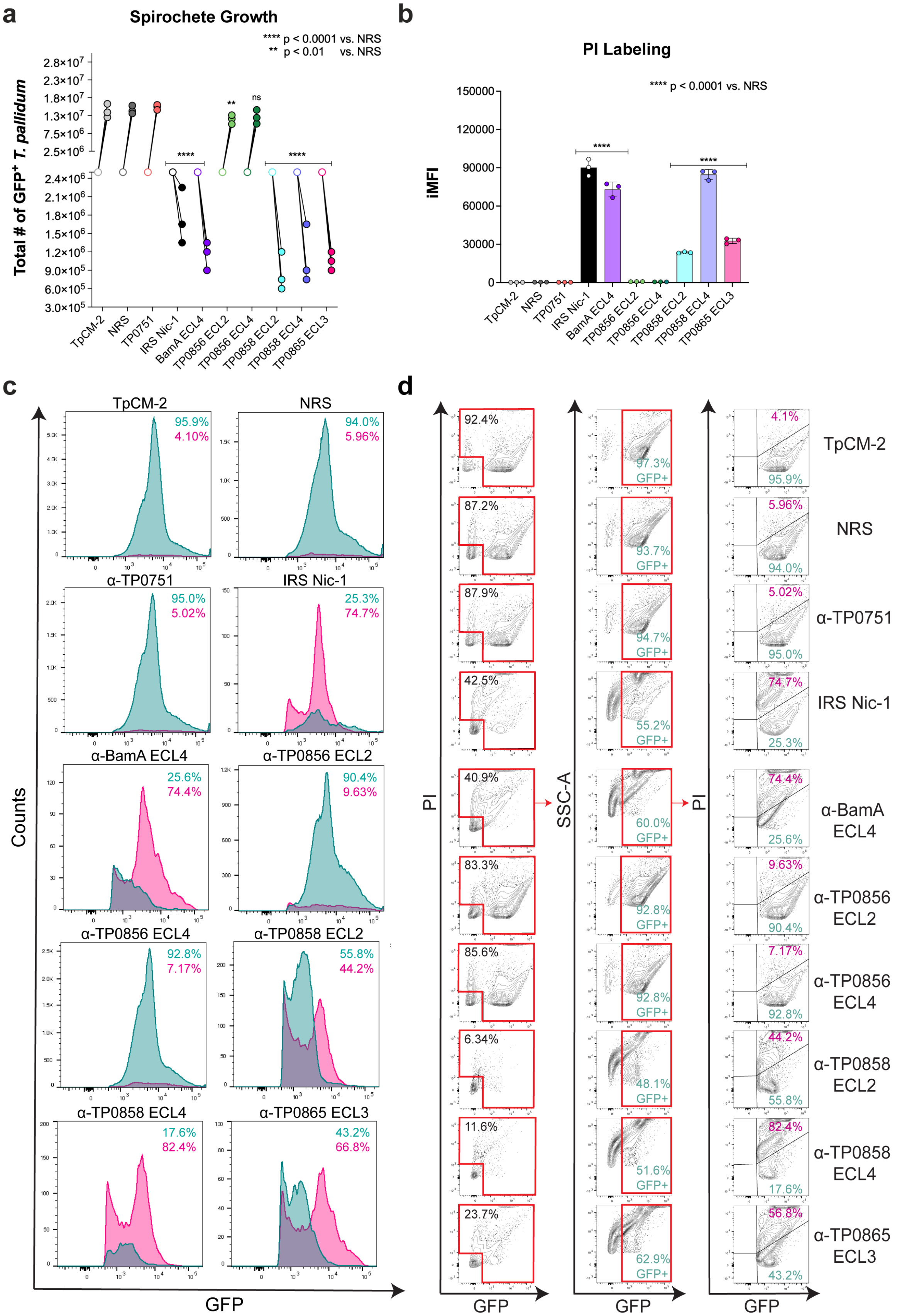
a
Spirochete Growth
**** p < 0.0001 vs. NRS
** p < 0.01 vs. NRS
Total # of GFP+ T. pallidum
TpCM-2
NRS
TP0751
IRS Nic-1
BamA ECL4
TP0856 ECL2
TP0856 ECL4
TP0858 ECL2
TP0858 ECL4
TP0865 ECL3
b
PI Labeling
**** p < 0.0001 vs. NRS
iMFI
c
Counts
GFP
d
PI
SSC-A
GFP
TpCM-2
NRS
α-TP0751
IRS Nic-1
α-BamA ECL4
α-TP0856 ECL2
α-TP0856 ECL4
α-TP0858 ECL2
α-TP0858 ECL4
α-TP0865 ECL3

**Figure S5 | Evaluation of antisera effects on *TPA* viability and OM integrity prior to intradermal infection of rabbits. (A)** Enumeration of total GFP-expressing *TPA* co-cultured with Sf1Ep cells in the presence of various antisera used prior to rabbit intradermal infection. Open symbols represent initial seeding densities; closed symbols indicate densities after 7 days of co-culture with corresponding antisera. **(B)** Representative flow cytometry histograms showing the proportion of $GFP^{+}$ *TPA* that are $PI^{-}$ (cyan) or $PI^{+}$ (magenta) following a 7-day incubation with indicated sera (10% final concentration). **(C)** Quantification of PI-labeled spirochetes calculated by iMFI for each condition. Panels a-b reflect the condition of the spirochete OM at the time of intradermal infection in rabbits. Data represent the mean ± SD from three technical replicates per condition. $^{**}p < 0.01$, $^{****}p < 0.0001$ vs. NRS. **(D)** Gating strategy used to assess OM disruption of *in vitro*-cultivated $GFP^{+}$ *TPA.*
